# Diabetes Prevention and Care Capacity at Urban Indian Health Organizations

**DOI:** 10.3389/fpubh.2021.740946

**Published:** 2021-11-26

**Authors:** Meredith P. Fort, Margaret Reid, Jenn Russell, Cornelia J. Santos, Ursula Running Bear, Rene L. Begay, Savannah L. Smith, Elaine H. Morrato, Spero M. Manson

**Affiliations:** ^1^Centers for American Indian and Alaska Native Health, Colorado School of Public Health, University of Colorado Anschutz Medical Campus, Aurora, CO, United States; ^2^Department of Health Systems, Management and Policy, Colorado School of Public Health, University of Colorado Anschutz Medical Campus, Aurora, CO, United States; ^3^Environmental Studies-Indigenous Sustainability Studies Program, Bemidji State University, Bemidji, MN, United States; ^4^Department of Population Health, School of Medicine and Health Sciences, University of North Dakota, Grand Forks, ND, United States; ^5^Parkinson School of Health Sciences and Public Health, Loyola University Chicago, Chicago, IL, United States; ^6^Department of Community and Behavioral Health, Colorado School of Public Health, University of Colorado Anschutz Medical Campus, Aurora, CO, United States; ^7^Department of Psychiatry, School of Medicine, University of Colorado Anschutz Medical Campus, Aurora, CO, United States

**Keywords:** sustainability, implementation science, organizational capacity, diabetes, Urban Indian Health Organization (UIHOs), American Indian and Alaska Native

## Abstract

American Indian and Alaska Native (AI/AN) people suffer a disproportionate burden of diabetes and cardiovascular disease. Urban Indian Health Organizations (UIHOs) are an important source of diabetes services for urban AI/AN people. Two evidence-based interventions—diabetes prevention (DP) and healthy heart (HH)–have been implemented and evaluated primarily in rural, reservation settings. This work examines the capacity, challenges and strengths of UIHOs in implementing diabetes programs.

**Methods:** We applied an original survey, supplemented with publicly-available data, to assess eight organizational capacity domains, strengths and challenges of UIHOs with respect to diabetes prevention and care. We summarized and compared (Fisher's and Kruskal-Wallis exact tests) items in each organizational capacity domain for DP and HH implementers vs. non-implementers and conducted a thematic analysis of strengths and challenges.

**Results:** Of the 33 UIHOs providing services in 2017, individuals from 30 sites (91% of UIHOs) replied to the survey. Eight UIHOs (27%) had participated in either DP (*n* = 6) or HH (*n* = 2). Implementers reported having more staff than non-implementers (117.0 vs. 53.5; *p* = 0.02). Implementers had larger budgets, ~$10 million of total revenue compared to $2.5 million for non-implementers (*p* = 0.01). UIHO strengths included: physical infrastructure, dedicated leadership and staff, and community relationships. Areas to strengthen included: staff training and retention, ensuring sufficient and consistent funding, and data infrastructure.

**Conclusions:** Strengthening UIHOs across organizational capacity domains will be important for implementing evidence-based diabetes interventions, increasing their uptake, and sustaining these interventions for AI/AN people living in urban areas of the U.S.

## Introduction

Urban American Indian and Alaska Native (AI/AN) people suffer a disproportionate burden of diabetes and cardiovascular disease (CVD). Compared to whites, urban AI/AN people are 20% more likely to die of heart disease and are three times more likely to die from diabetes (1). Many urban AI/AN people receive care at Urban Indian Health Organizations (UIHOs), which provide culturally appropriate primary health care services. While more than 70% of AI/AN people live in cities, <1% of the Indian Health Service (IHS) budget—the major federal agency charged with caring for Native peoples in the US—is dedicated to addressing their health needs (2).

Recognizing the need for expanded health services, the United States Congress established the Special Diabetes Program for Indians (SDPI) in 1997. Within that program, two IHS demonstration projects were initiated to translate evidence-based practices for diabetes and CVD prevention to AI/AN communities and people (3). The first project, SDPI-Diabetes Prevention (DP), modeled after the National Institute of Health's Diabetes Prevention Program (DPP) (4), aimed to reduce and delay the onset of diabetes among pre-diabetic individuals. The second project, SDPI-Healthy Heart (HH) focused on intensive case management activities to reduce CVD risk factors in individuals with diagnosed diabetes. SDPI's national competitive grant program encouraged existing SDPI recipients to compete for additional funds and provided the opportunity to participate in a collaborative process to develop and implement DP or HH projects.

Implementation and evaluation of the SDPI-DP demonstration project reported similar success to the original DPP; crude diabetes incidence was 4.0% per year for participants and on average participants lost 9.6 pounds after participating in the 16 lifestyle balance classes (5). SDPI-HH also showed that intensive case management on multiple CVD risk factors resulted in improvements in the primary outcomes: blood glucose, blood pressure, and lipid control. A1C levels decreased 0.2% on average, systolic and diastolic blood pressure both improved, and the largest measurable effect was reduction in low-density lipoprotein (LDL) cholesterol from baseline to 1 year (−5.29 mg/dL) (6). Emphasis on cultural adaptation was recognized as being central to these experiences (7). Building on this initial success, IHS transitioned all funding for the DP and HH initiatives to the community-directed programs, thereby institutionalizing this initiative within its broader program. SDPI developed toolkits based on the initial experiences to be used by other settings for future implementation.

**Figure 1:**
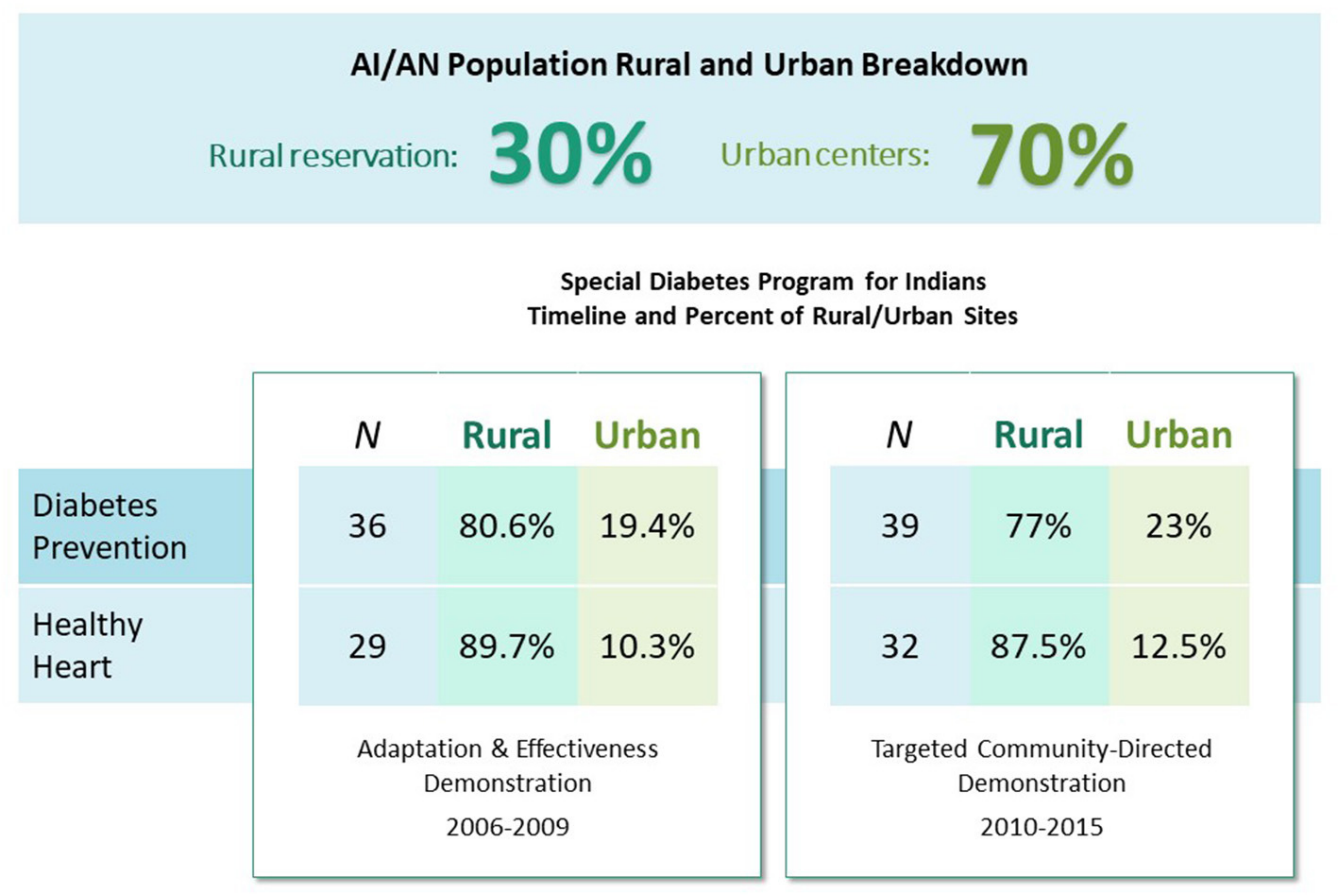
Rural and urban diabetes demonstration program implementing sites: timeline and AI/AN population percentages.

Figure 1 summarizes the evolution of the IHS DP and HH programs by medical care delivery setting and stage of dissemination and implementation. The figure shows that early SDPI-DP and HH implementation occurred in predominantly rural, reservation settings. To extend the reach of these programs and complete national scale-up to locations where the majority of AI/AN people reside, dissemination needs to be adapted for Urban Indian Health Organization (UIHO) settings.

Without an emphasis on context, the uptake of evidence-based programs is slow, reach tends to be limited, and sustained implementation is a challenge (8–10). Even if providers and administrators learn about successful programs, they may have difficulty implementing them, including those tailored specifically to the population they serve. UIHOs, as compared to rural reservation settings, receive a smaller portion of their funding from the IHS and depend on Medicaid, grants, and other sources to offer services to a patient population with a high level of need. Low-resource safety net settings often require capacity building efforts to take on and sustain new programs (11).

To further support national expansion and sustained implementation of evidence-based diabetes prevention and care programs in UIHO settings, our study aimed to assess organizational capacity domains relevant to the implementation and sustainment of these programs, gauge familiarity with DP and HH programs, and capture perceived challenges and opportunities.

## Methods

### Survey Design and Data Collection

We conducted a cross-sectional analysis of an original 75-question survey for UIHOs aimed at assessing organizational capacity and aspects of diabetes prevention and care that would benefit from investment and strengthening. From the literature, we identified eight domains of organizational capacity for assessing public health systems and services: fiscal and economic resources, workforce and human resources, physical infrastructure, inter-organizational relations, informational resources, system boundaries and size, governance and decision-making structure, and organizational culture (12). The survey was created in SurveyMonkey which included an assessment of diabetes prevention and care program experience that drew from a diabetes care coordination survey (13); SDPI familiarity; the eight domains of public health services and systems organizational capacity (12); and strengths and challenges that UIHOs face in implementing their work. Generally, there were three types of responses: yes, no, other, and don't know; five-point Likert scale; and open-ended responses.

UIHOs were identified using the Urban Indian Health Program Profiles at the Urban Indian Health Institute website (14). Thirty-three UIHOs were identified as actively serving clients during 2017 and were classified as comprehensive clinics (providing direct primary care for at least 40 h per week), limited clinics (providing direct primary care for under 40 h per week), and outreach and referral (no direct service on site, patients externally referred) (15). We categorized UIHOs as having been implementers or non-implementers, using the DP and HH grant recipient list.

Prior to administering the survey, we assembled a list of potential contacts at each organization in the following employment categories: chief executive officer/organizational director/chief operating officer; financial director/chief financial director/fiscal manager; clinic manager/director of health services/chief medical officer; diabetes program coordinator/diabetes project director; and information technology specialist/lead. Not all UIHOs had the five employment categories and some positions were vacant. The contact list was compiled through information found on organizational websites, and through phone calls and emails to organizations. In addition, our local partnering UIHO in Denver, CO wrote a letter inviting other UIHOs to participate in the survey which was sent via email with a link to the survey. The survey was determined “not human subject research” by the Colorado Multiple Institution Review Board.

Data was collected from April 13 through May 26, 2017. We sent weekly emails to follow-up with non-responders and those with incomplete surveys. Thirty organizations completed the survey. One organization declined participation indicating they were unable to participate due to management changes. Two UIHOs were non-responsive to the five follow-up attempts to seek survey participation.

Financial data including revenue, expenses, and revenue source came from the Internal Revenue Service (IRS) form 990, which is publicly available. Completion of this form is required for all tax-exempt organizations, including non-profits. All UIHOs filed with the IRS as 501(c)(3) non-profit organizations and completed the form 990. Completed forms were downloaded from ProPublica's Nonprofit Explorer database (16). We used data for either fiscal year 2016 or 2017 depending on the UIHO's filing period and which filing included April to May 2017.

### Data Analysis

We summarized the questions by organization to describe characteristics at the organization level. When responses differed for respondents within an organization, we used the response of the respondent most likely to know the answer based on the relevance of their position to the question. If all respondents either did not answer the question or replied, “don't know,” then the variable was left blank in the analysis. For ease of interpretation, Likert responses were dichotomized into “Agree” (3–5) or “Disagree” (1, 2) and if any respondents indicated agreement, the organization's response was classified as “Agree.”

We summarized data in descriptive tables including the frequencies for categorical responses and reported either mean and standard deviation (SD) or median and interquartile range (IQR) for continuous variables. Descriptive statistics were categorized by whether the UIHO had implemented or not implemented either program. As the distribution for questions across implementation category was sparse, we used Fisher's exact tests and Kruskal-Wallis exact tests for binary and continuous variables, respectively. Among the 75 original questions, we selected 57 across all eight domains for reporting. We chose not to include questions if they did not offer substantially new insight into organizational capacity domains or for which there appeared to be interpretation heterogeneity. Most questions that were not included were related to specific aspects of the diabetes registry and modes of communication. In addition, we did not include some questions that captured baseline attributes of physical space (e.g., having private work stations or space to meet privately with patients) as they refer to more basic conditions that all of the interviewed UIHO sites met. Interested readers may contact the authors for the full set of questions. Open-ended questions about strengths and challenges were analyzed for themes among all the survey respondents. We report the themes along with representative quotes or phrases.

## Results

**Figure 2:**
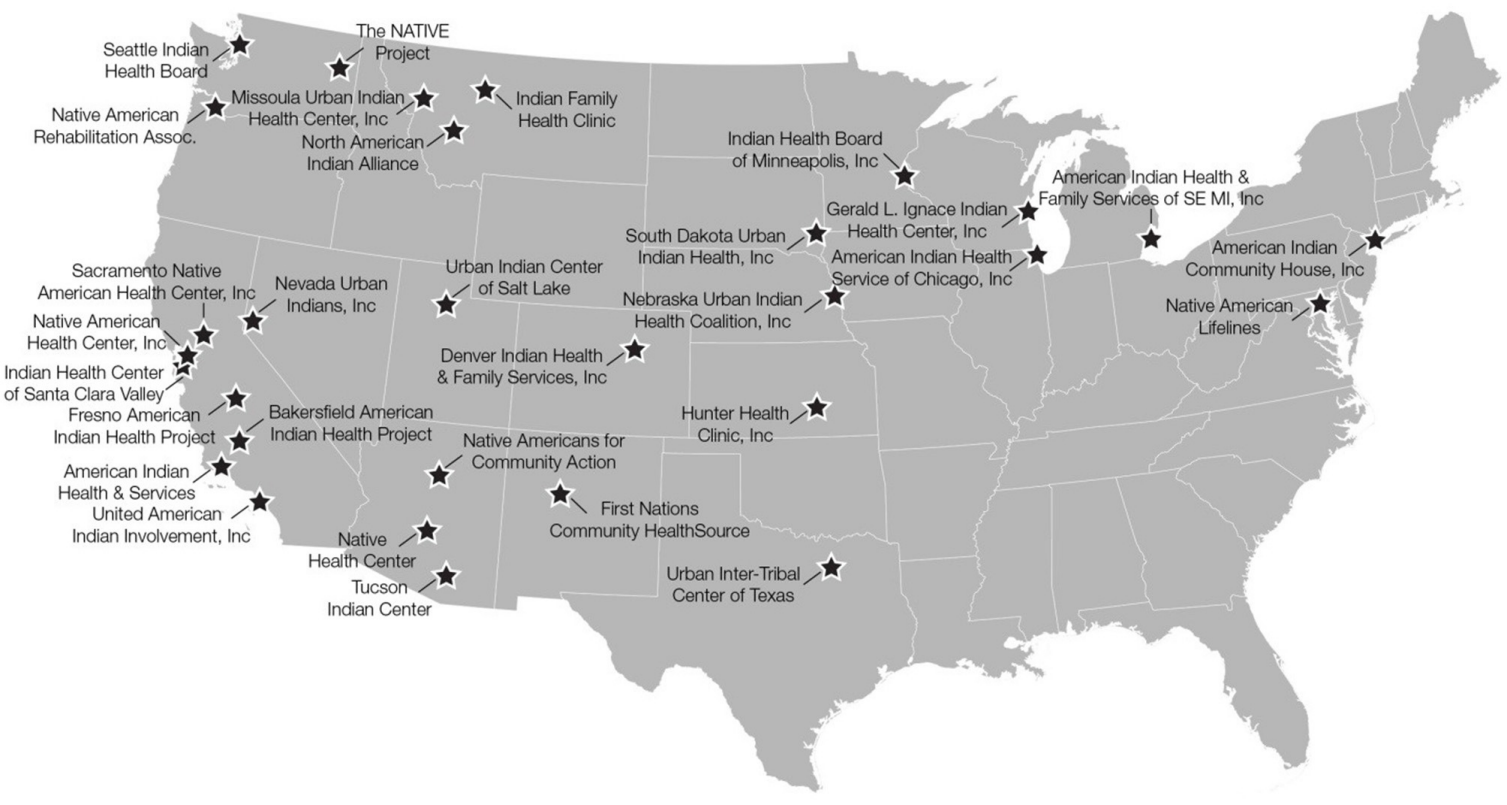
Urban Indian Health Organization diabetes capacity survey participants.

Of the 33 UIHOs providing services in 2017, 30 (91%) across 19 states participated in the survey about diabetes organizational capacity (see Figure 2 for locations). Among the UIHOs, we identified 117 individuals representing the different roles of interest, of which a total of 72 (62%) responded to the survey. The breakdown of the type of respondents was CEO (17.8%), Fiscal (13.7%), Clinical lead (30.1%), Diabetes-specific (28.8%), Information Technology (IT) (9.6%). Of the 30 UIHOs, 8 (27%) participated in either DP (*n* = 6) or HH (*n* = 2).

The UIHOs varied substantially in terms of key characteristics (see Table 1). Total annual revenue ranged from ~$700,000 to over $28 million. Size of the organization with respect to total population served also had a large range: from 200 to over 25,000 people. The oldest UIHO dates to 1963 and four have been in operation for fewer than 20 years, having opened their doors after 2000.

**Table 1:** Central features of Urban Indian Health Organizations.

	**Average, most common response, or N (where noted)**	**Range**
Years in operation, *N*		Year the UIHO opened:
10–20	5	1963–2008
20–30	0	
30–40	3	
40 or more	21	
Population served	5,638	201 to more than 25,000
Service offering	Primary care	Behavioral health; dental; optical; health promotion; social services; cultural programs and community integration
FQHC, *N*	10	–
Number of staff	69	7–254
Number of physicians (MDs/DOs)	3	0–19
Number of non-physician clinicians (PAs/NPs)	3	0–12
Total revenue^a^	7.1 million	$695,016 to $28.2 million
Operating margin^a^	$382,053	-$203,323 to $2.7 million
Revenue from government grants^a^	66.2%	0–99.5%
Revenue from program services^a^	31.7%	0–99%

*Source data: Self-reported survey of Urban Indian Health Organizations, N = 30 (2017). FQHC, Federally-Qualified Health Center; MD, Doctor of Medicine; DO, Doctor of Osteopathy; PA, Physician Assistant; NP, Nurse Practitioner*.

a *Data came from the IRS Form 990 for whichever reporting period contains April-May 2017*.

The range of services provided by the different UIHOs varied with most providing primary care often with additional services including dental, optical, and behavioral health. Several UIHOs offer additional services relevant to diabetes including health promotion, exercise classes, and nutritional support. Some UIHOs also included social services aimed at addressing the social determinants of health and facilitating cultural connections and community integration including transportation, voter registration, employment preparation and referrals, ceremonies, arts and crafts, and afterschool programs. Nineteen of the 30 UIHOs were classified as comprehensive clinics, including five of the DP and both HH implementers; five were classified as limited clinics; and six, all non-implementers, were classified as outreach and referral organizations. One third of the UIHOs were federally-qualified health centers that receive funds from the US Health Resources and Services Area (HRSA) Health Center Program to provide primary care services in underserved areas.

Almost all organizations reported offering services or programs for diabetes prevention and treatment. One implementer only offered treatment programs and the others offered both prevention and treatment programs. Among non-implementers, 90% offered prevention and 86% treatment services or programs. Respondents reported a high level of familiarity with SDPI, HH and DP (100% for implementers and 90% for non-implementers). All but three of the organizations reported viewing the DP and HH toolkits.

Table 2 presents item ratings for each of the organizational capacity domains comparing UIHOs that did not implement DP or HH, with UIHOs that implemented the programs. With respect to workforce and human resources, implementers reported having significantly more staff (clinical and non-clinical) than non-implementers (117.0 vs. 53.5; *p* = 0.02). DP and HH implementers, compared to non-implementers, had significantly more physicians (4.3 vs. 2.2; *p* = 0.03) and non-physician clinicians (e.g., nurse practitioners and physician assistants; 4.1 vs. 2.1; *p* = 0.03). 62.5% of implementers (and 83% of DP implementers) had a certified diabetes educator (CDE) compared to 36% of non-implementers; across all UIHOs, fewer than half had a CDE. Only 55% of non-implementers had a registered dietitian compared to 100% of both DP and HH implementers (*p* = 0.03). Nearly 75% of all UIHOs reported having vacancies that affect patient care.

**Table 2:** Comparison of implementers vs. non-implementers by organizational capacity domain.

	**Non-implementers** ***N*** **= 22**		**SDPI implementers** ***N*** **= 8**		**All** ***N*** **= 30**		**Significant difference (*p*-value)^a^**
**Workforce and human resources**							
Number of staff employed, Mean SD	53.5	63.8	117.0	86.9	68.8	73.7	0.02
Number of physicians (MDs/DOs) employed, Mean SD	2.2	4.1	4.3	4.5	2.8	4.2	0.03
Number of non-physician clinicians (PAs/NPs) employed, Mean SD	2.1	2.8	4.1	2.9	2.7	2.9	0.03
Certified diabetes educator, *N* %	8	36.4%	5	62.5%	13	43.3%	0.24
Registered dietitian*, N* %	12	54.6%	8	100.0%	20	66.7%	0.03
Physical activity specialist*, N* %	8	36.4%	3	37.5%	11	36.7%	1.00
Health educator/Lifestyle counselor, *N* %	14	66.7%	7	87.5%	21	72.4%	0.38
Community health worker, *N* %	13	59.1%	6	75.0%	19	63.3%	0.67
Nurses (RN, BSN, or MSN), *N* %	15	68.2%	8	100.0%	23	76.7%	0.14
Public health or epidemiology, *N* %	8	36.4%	2	28.6%	10	34.5%	1.00
Existing vacancies that affect patient care, *N* %	16	72.7%	6	75.0%	22	73.3%	1.00
**Fiscal and economic resources**							
Total revenue in thousands^b^, Median IQR	2,519	1,433–7,169	9,830	5,807–20,453	3,378	1,825–10,939	0.01
Revenue less expenses in thousands^b^, Median IQR	112	2–312	244	33–880	112	26–576	0.53
Operating margin^b^, Median IQR	4.1%	0.2–10.7%	1.3%	0.9–6.0%	3.7%	0.8–10.7%	0.73
Percent of revenue from government grants^b^, Median IQR	75.7%	52.4–91.8%	56.8%	39.0–69.9%	68.8%	52.4–91.4%	0.22
Percent revenue from program services^b^, Median IQR	16.2%	3.7–57.6%	37.2%	27.4–57.7%	23.3%	3.7–57.6%	0.24
Available funding for diabetes prevention and care, *N* %	17	77.3%	7	87.5%	24	80.0%	1.00
Funding specifically dedicated for diabetes prevention and care, *N* %	18	81.8%	6	75.0%	24	80.0%	0.65
Sufficient funding for my organization's diabetes programs, *N* %	9	40.9%	4	50.0%	13	43.3%	0.70
**Physical infrastructure**							
Space to facilitate group education classes on-site, *N* %	21	95.5%	8	100.0%	29	96.7%	1.00
Equipment for nutrition education (e.g., kitchen), *N* %	15	68.2%	6	75.0%	21	70.0%	1.00
Physical activity equipment on-site, *N* %	13	59.1%	5	62.5%	18	60.0%	1.00
Laboratory collection and testing equipment, *N* %	17	77.3%	8	100.0%	25	83.3%	0.29
Pharmacy, *N* %	4	18.2%	3	37.5%	7	23.3%	0.34
**Data and informational resources**							
Use an EHR system, *N* %	22	100.0%	8	100.0%	30	100.0%	NA
Reliable access to the internet, *N* %	22	100.0%	8	100.0%	30	100.0%	NA
Uses paper charts, *N* %	15	68.2%	4	50.0%	19	63.3%	0.42
Current registry that identifies and tracks patients with type 2 diabetes, *N* %	21	95.5%	7	87.5%	28	93.3%	0.47
Ability to systematically identify patients who have pre-diabetes, *N* %	16	76.2%	8	100.0%	24	82.8%	0.28
EHR provides historical reports on patients' lab results, *N* %	16	72.7%	7	87.5%	23	76.7%	0.64
EHR provides reports on preventive health measures (e.g., physical activity, diet), *N* %	16	72.7%	6	75.0%	22	73.3%	1.00
EHR is able to generate reminders about diabetes care targeted at the provider, *N* %	16	72.7%	6	75.0%	22	73.3%	1.00
**System boundaries and size**							
UIHO is a FQHC, *N* %	5	22.7%	5	62.5%	10	33.3%	0.08
Approximate number of patients served in a fiscal year, Mean SD	4,540	6,627	8,382	7,644	5,638	7,013	0.04
Approximate number of patients receiving care with type 2 diabetes^c^, Mean SD	272	280	593	513	362	378	0.05
Percentage of patients that identify as AI/AN^c^, Mean SD	71.9%	33.5%	53.9%	34.4%	67.2%	34.0%	0.15
Percentage of patients that prefer a language other than English^c^, Mean SD	7.0%	11.1%	13.7%	24.3%	8.7%	15.3%	0.48
Location of my facility is accessible to patients, *N* %	22	100.0%	8	100.0%	30	100.0%	NA
Assist patients with transportation costs, *N* %	18	81.8%	8	100.0%	26	86.7%	0.55
Agreements with other community-based organizations for patients to use relevant services not available at the clinic (e.g., nutrition/meals, physical activity, housing, financial needs, etc.), *N* %	17	77.3%	7	87.5%	24	80.0%	1.00
Policies and procedures are in place for identifying specialty referrals, *N* %	22	100.0%	8	100.0%	30	100%	NA
**Inter-organizational relationships, governance and decision-making structure**							
Organization is in regular communication with the Indian Health Service, *N* %	21	95.5%	8	100.0%	29	96.7%	1.00
Organization has a working relationship with its Board of Directors, *N* %	21	95.5%	7	87.5%	28	93.3%	0.47
Community stakeholders participate in the organization's decision-making process, *N* %	15	68.2%	8	100.0%	23	76.7%	0.14
Organization uses patient/family feedback to improve the quality of our services, *N* %	22	100.0%	8	100.0%	30	100.0%	NA
**Organizational culture**							
Staff at my organization have high morale, *N* %	18	81.8%	8	100.0%	26	86.7%	0.55
Clinic leadership is supportive of diabetes care and prevention services, *N* %	22	100.0%	8	100.0%	30	100.0%	NA
Most staff are AI/AN, *N* %	16	72.7%	4	50.0%	20	66.7%	0.38
Programs and departments often collaborate, *N* %	22	100.0%	8	100.0%	30	100.0%	NA
Organization leadership is visible and accessible, *N* %	21	95.5%	8	100.0%	29	96.7%	1.00
Organization is trusted by patients, *N* %	21	95.5%	7	87.5%	28	93.3%	0.47
Family members/care givers are encouraged to participate in health education activities, *N* %	21	95.5%	7	87.5%	28	93.3%	0.47

*SD, Standard Deviation; IQR, Inter-quartile range; MD, Doctor of Medicine; DO, Doctor of Osteopathic Medicine; PA, Physician Assistant; NP, Nurse Practitioner; RN, Registered Nurse; BSN, Bachelor of Science in Nursing; MSN, Master of Science in Nursing; EHR, Electronic Health Record; FQHC, Federally Qualified Health Center*.

a *Fisher's exact tests and Kruskal-Wallis exact tests were used for identifying significant differences across groups for binary and continuous variables, respectively. Tests were not computed for questions for which all UIHOs answered identically*.

b *Data come from the IRS Form 990 for whichever reporting period contains April-May 2017*.

c *Question had 3–5 organizations with a missing response*.

For fiscal and economic resources, DP and HH implementers had significantly larger budgets than non-implementers, ~$10 million of total revenue for implementers compared to $2.5 million for non-implementers (*p* = 0.03). The percentage of revenue from government grants was greater for non-implementers (76%) than for implementers (57%) and percentage of revenue from program services was greater for implementers than non-implementers (37 vs. 16%). While 80% of UIHOs reported having dedicated funds for diabetes prevention and care, less than half of both implementers and non-implementers responded that funding was sufficient.

For the remaining domains, no significant differences were reported for implementers and non- implementers. For physical infrastructure, all but one UIHO reported having space to facilitate group education classes on-site. A similar percentage of implementer and non-implementer UIHOs (68 and 67%, respectively), had equipment for nutrition education such as a kitchen. A smaller percentage (60% of all UIHOs) has on-site physical activity equipment. Only 23% of UIHOs had an on-site pharmacy. For data and informational resources, all UIHOs reported using electronic health records; ~75% were able to report on preventive measures and generate clinical care reminders for providers. For this survey, five UIHOs did not report the current number of patients served with diabetes; this type of tracking appeared to be difficult, as there was a notable lack of numbers reported, especially by clinical staff who responded to the survey. For the system boundaries and size domain, UIHOs noted patients face multiple social, economic, housing, and transportation barriers that may not allow them to prioritize health care. Organizational culture was rated as a strength, with similar findings for DP and HH implementers as well as non-implementers.

### Reported Strengths and Challenges

In response to open-ended questions about strengths and challenges, respondents identified the following key strengths related to workforce and human resources: dedication of staff; CDEs, dietitians or other staff who were very engaged in diabetes programs; and a high percentage of AI/AN staff. Team-work and collaboration between staff members were highlighted as important such as when teams “participate in warm hand-offs to be able to provide point-of-care education around diabetes prevention and care.” UIHOs reported the range of services that they offer on-site as a strength. One UIHO stated: “We have the full spectrum of integrated care in regards to physical health, behavioral health, and a wellness center, all under one location.” However, others referred to space restrictions (limited exam rooms, no space for cooking demonstrations, no fitness facility) as challenges to implementing diabetes programs. Organizations mentioned having good rapport with the community, community-driven work, and being responsive to community need by having flexible hours, culturally-sensitive education/prevention for Native Communities, and family-orientated outreach activities. One UIHO stated: “We have a large community that relies on the limited services we provide and have high usage/retention in programs.” Key challenges included: high staff turnover and the difficulty of recruiting and retaining staff for specific positions including CDEs and nurses. UIHOs reported inconsistent funding and dependency on grants to fund many programs as key challenges. Some programs stated that when grant funds run out, they had to let staff go or cut back on services.

## Discussion

UIHOs are an important network of safety net institutions providing diabetes prevention and care for urban AI/AN people across the country. There is a substantial range in capacity, service offerings, and funding for the 30 UIHOs that participated in this study. Overall, they have good physical infrastructure, dedicated leadership and clinic staff, and strong community relationships.

Our findings indicate that the top two capacity domains relevant to implementation of evidence-based diabetes prevention and care programs were: (1) workforce and human resources and (2) financial and economic resources. Data and informational resources were a third priority domain; tracking participation and outcomes was required in the implementation experience and is important for ongoing assessment. Implementers had on average nearly twice as many staff members than non-implementers, and implementers had significantly more clinical staff. For specific positions that are key to implementing the programs (CDE, registered nurse, or lifestyle educator for DP), if UIHOs do not have trained staff onsite, they have to hire or train staff to take on the tasks of implementing DP. Notably, implementers had significantly larger total revenue than non-implementers, and lower percent revenue from government grants and higher percent revenue from program services (e.g., patient care). This flexibility in funding may have positioned them better to adopt DP and HH compared to non-implementers. The minimum eligibility criteria to apply for DP and HH were defined as the ability to implement the programs and to track individual-level participant improvement to establish an evidence base. Presumably, a large portion of the UIHOs did not meet the minimum criteria and would likely need to strengthen their capacity in order to implement one or both of the programs in the future; as such resources dedicated to creating the necessary conditions for implementation will be important.

According to the open-ended questions, key organizational areas identified as priorities to strengthen in order to enable broader uptake of effective and culturally appropriate diabetes programs are: health workforce training, accreditation, and hiring to increase lifestyle counseling, diabetes self-management education, and diabetes clinical care capacity; improvement of recruitment and retention of staff; assuring that data infrastructure allows for UIHO staff to track patients with diabetes and pre-diabetes who are eligible to participate in DP and HH programs; and sufficient and consistent funding. The concern that UIHOs expressed about sustainable funding is closely linked to human resources, as trained staff can no longer be supported when grant funding ends. In the area of human resources, UIHOs face difficult decisions about whether to prioritize building capacity internally or hiring new staff. With respect to data capacity, the ability to track patients and community members who are eligible to participate in evidence-based diabetes prevention and care programs is critical for assessing the program's reach and representativeness, and for ensuring equitable delivery.

Our study has several important strengths. All but three of the UIHOs that were identified as actively offering services in the country in 2017 participated; as such, it provides a comprehensive view of diabetes prevention and care capacity for AI/AN-serving safety net organizations in urban areas nationwide. The study joins others in filling the research gap on services and health needs of urban AI/AN people (17). The study emphasized organizational capacity, a topic important for uptake of evidence-based diabetes prevention and care programs. Finally, open-ended responses related to strengths and challenges revealed common priorities that may serve as a collective roadmap for UIHO organizational capacity building in diabetes prevention and care.

This study also has a few limitations. Participation in the survey was not the same across the different UIHOs (some sites had up to five respondents whereas others only had one). Not all respondents replied to every question. It was difficult to reconcile differing responses within an organization for certain questions such as service offerings and the population served by the organization. Due to the small number of UIHOs and few implementers, we relied on non-parametric tests to examine statistical differences; these tests do not allow for adjustment by possible confounding factors, such as size. Finally, questions focused on the capacity of organizations without addressing the broader policy context. Questions about local, state and national level factors that influence diabetes prevention and care within UIHOs would have offered additional insight.

Our study identifies multiple topics that will benefit from additional research. We propose to update and streamline the survey for future use; a version with standard benchmarks and priority areas could encourage UIHOs to operationalize change. The Assessment of Chronic Illness Care instrument, which assesses the domains of the Chronic Care Model, serves as a tool for teams to assess their current level of implementation, areas for improvement, and serves as a guide for future implementation (18). Incorporating newly-developed measures may also strengthen the survey in the future (19). It will be important to make an effort to update the UIHOs potentially eligible as implementers, given that the total number of UIHOs varies according to source and has increased over time. We identified several topics that would be beneficial to expand in a future version of the survey. For example, with respect to human resources, while important to enumerate staff with specific training and skills, the qualitative portion of the survey pointed to the importance of capturing other characteristics including an emphasis on teamwork and a commitment to serving the AI/AN community. Future analyses with complementary data could explore questions such as whether there was a baseline administrative capacity for implementing programs. Additional analytic approaches such as factor analysis or system dynamics could be conducted in the future, to better understand aspects of the domains and the dynamic system in which diabetes care is delivered (20–22).

There is an increased call in the field of dissemination and implementation (D&I) for assessing context to understand the conditions for improvement (23, 24). Recognizing the diverse levels of organizational capacity captured by the survey, we recommend carrying out additional research into the dissemination and uptake of evidence-based diabetes interventions to UIHOs. Simultaneous roll-out within UIHOs with differing levels of capacity would allow for a better understanding of the influence of organizational capacity on the implementation and scale-up of diabetes programs in these diverse settings. Some scholars in the field of implementation science call for the explicit comparison of dimensions of context in the design stage of research studies to increase our understanding of the influence of context on implementation (25). Surveys such as the one used for this study can identify priority dimensions of context for such studies. Documenting adaptations that are needed to facilitate increased uptake, equitable delivery, and sustainment of DP and HH in UIHO settings will also contribute to the field (26–29).

### Policy and Practice Implications

Our study points to several priority policy and practice implications. For the financial and economic resources domain, UIHOs referred to the unpredictable and inconsistent nature of grant funds as a challenge. Implementers had a higher percentage of revenue program services than grants. This may have allowed some UIHOs more flexibility in considering to be implementers. A concrete opportunity for expanding stable funding for DP within UIHOs is through Medicaid reimbursement. Funding from the Centers for Medicaid and Medicare Services programs—and in particular Medicaid—has been recognized as an important source for AI/AN people (30). In 2017, 43% of UIHO patients were insured by Medicaid (31), but only about half of the states where UIHOs are located have Medicaid programs that reimburse for the DPP. To improve funding for DP within UIHOs, states across the country should make the DPP a Medicaid covered service. Another opportunity to increase the number of sites that are in a position to take on the implementation of DP and HH, or other evidence-based diabetes interventions, would be for funders to support capacity building in addition to implementation; funders could support a needs assessment to identify capacity gaps, in addition to supporting program implementation.

On a national scale, it is important to support initiatives to train and retain staff involved in diabetes service implementation, including registered dietitians and CDEs (32), to fill the needs of UIHOs over the long term. Other positions such as pharmacists and primary care team members may be central to program implementation too (33). Established agreements and practicums between University health professional programs and UIHOs could help facilitate future placement of newly trained providers; training programs that emphasize the historical and social context of the population served by UIHOs are especially promising (34).

While infrastructure of UIHOs is a recognized strength, many do not have the full range of on-site infrastructure to support patients in the prevention and self-management of diabetes (kitchen facilities, on-site gym equipment, in-house pharmacy and laboratory services). Aligning with and referring to other programs and resources may be a way to facilitate the uptake of diabetes programs such as establishing partnerships with public recreation centers or gyms, nutrition education programs, and nearby pharmacies and laboratories.

## Conclusion

UIHOs offer an important opportunity to provide culturally appropriate evidence-based diabetes prevention and care services to the millions of AI/AN people who live in cities. UIHOs have a range of organization capacity, services, and size of population served. UIHOs recognize their dedicated leadership and staff, services that address basic needs, good relationships with the community, and existing diabetes programs as key strengths. With consistent funding to sustain diabetes programs, sufficient staffing and mechanisms for retention, and data infrastructure support, UIHOs promise to contribute substantially to increasing the delivery of appropriate diabetes prevention and care for AI/AN communities and address a pressing health disparity.

## Data Availability Statement

The raw data supporting the conclusions of this article will be made available by the authors, without undue reservation.

## Author Contributions

MPF, JR, URB, EHM, and SMM designed the survey and initially conceptualized the paper. URB, RLB, and SLS captured and summarized the survey data. MPF, MR, and CJS led the analysis. MPF and MR took the lead in writing an initial draft. All authors contributed to reviewing, revising, and rewriting sections of this article.

## Funding

MPF and MR received funding from the National Institutes of Health/National Heart, Lung and Blood Institute and MPF and SMM received funding from the National Institutes of Health/National Diabetes and Digestive and Kidney Diseases. The funders were not involved in the study design, collection, analysis, interpretation of data, the writing of the article or the decision to submit it for publication.

## Conflict of Interest

SLS is employed by the company JSI Research & Training Institute, Inc. and was employed by the Centers for American Indian and Alaska Native Health, Colorado School of Public Health, during the time that the work presented in this article was conducted. The remaining authors declare that the research was conducted in the absence of any commercial or financial relationships that could be construed as a potential conflict of interest.

## Publisher's Note

All claims expressed in this article are solely those of the authors and do not necessarily represent those of their affiliated organizations, or those of the publisher, the editors and the reviewers. Any product that may be evaluated in this article, or claim that may be made by its manufacturer, is not guaranteed or endorsed by the publisher.

## References

[1] Jacobs-Wingo JL Espey DK Groom AV Phillips LE Haverkamp DS. Causes and disparities in death rates among urban American Indian and Alaska Native populations, 1999-2009. Am J Public Health. (2016) 106:906–14. 10.2105/AJPH.2015.303033 26890168 PMC4985112

[2] Yuan NP Bartgis J Demers D. Promoting ethical research with American Indian and Alaska Native people living in urban areas. Am J Public Health. (2014) 104:2085–91. 10.2105/AJPH.2014.302027 25211730 PMC4202957

[3] Indian Health Service. (2020). SDPI website. Available online at: https://www.ihs.gov/sdpi/about/ (accessed December 18, 2020).

[4] Diabetes Prevention Program (DPP) Research Group. The Diabetes Prevention Program (DPP): description of lifestyle intervention. Diabetes Care. (2002) 25:2165–71. 10.2337/diacare.25.12.2165 12453955 PMC1282458

[5] Jiang L Manson SM Beals J Henderson WG Huang H Acton KJ. Translating the diabetes prevention program into American Indian and Alaska native communities: results from the special diabetes program for Indians diabetes prevention demonstration project. Diabetes Care. (2013) 36:2027–34. 10.2337/dc12-1250 23275375 PMC3687272

[6] Moore K Jiang L Manson SM Beals J Henderson W Pratte K. Case management to reduce cardiovascular disease risk in American Indians and Alaska Natives with diabetes: results from the special diabetes program for Indians healthy heart demonstration project. Am J Public Health. (2014) 104:e158–64. 10.2105/AJPH.2014.302108 25211728 PMC4202936

[7] Thornton PL Kumanyika SK Gregg EW Araneta MR Baskin ML Chin MH. New research directions on disparities in obesity and type 2 diabetes. Ann NY Acad Sci. (2020) 1461:5–24. 10.1111/nyas.14270 31793006 PMC7159314

[8] Brownson RC Colditz GA Proctor EK. Dissemination and Implementation Research in HEALTH: Translating Science to Practice. Second Edition. New York, NY: Oxford University Press (2018). 10.1093/oso/9780190683214.001.0001

[9] Green LW Ottoson JM García C Hiatt RA. Diffusion theory and knowledge dissemination, utilization, and integration in public health. Annu Rev Public Health. (2009) 30:151–74. 10.1146/annurev.publhealth.031308.100049 19705558

[10] Shelton RC Cooper BR Stirman SW. The sustainability of evidence-based interventions and practices in public health and health care. Annu Rev Public Health. (2018) 39:55–76. 10.1146/annurev-publhealth-040617-014731 29328872

[11] Moore SL Fischer I Havranek EP. Translating health services research into practice in the safety net. Health Serv Res. (2016) 51:16–31. 10.1111/1475-6773.12341 26646189 PMC4722217

[12] Meyer A-M Davis M Mays GP. Defining organizational capacity for public health services and systems research. J Public Health Manag Pract. (2012) 18:535–44. 10.1097/PHH.0b013e31825ce928 23023278

[13] Weeks DL Polello JM Hansen DT Keeney BJ Conrad DA. Measuring primary care organizational capacity for diabetes care coordination: the diabetes care coordination readiness assessment. J Gene Internal Med. (2014) 29:98–103. 10.1007/s11606-013-2566-2 23897130 PMC3889951

[14] Urban Indian Health Institute. Community Health Profile: National Aggregate of Urban Indian Health Program Service Areas. (2016). Available online at: https://www.uihi.org/urban-indian-health/urban-indian-health-organization-profiles/ (accessed December 30, 2020).

[15] Indian Health Service. Office of Indian Health Programs Strategic Plan: 2017-2021. (2021). Available online at: https://www.ihs.gov/sites/urban/themes/responsive2017/display_objects/documents/IndianHealthServiceOfficeofUrbanIndianHealthProgramsStrategicPlan.pdf (accessed July 13, 2021).

[16] ProPublica. ProPublica's Nonprofit Explorer Database. (2020). Available online at: https://projects.propublica.org/nonprofits/ (accessed June 8, 2020).

[17] James RD West KM Claw KG EchoHawk A Dodge L Dominguez A. Responsible research with Urban American Indians and Alaska Natives. Am J Public Health. (2018) 108:1613–6. 10.2105/AJPH.2018.304708 30359103 PMC6236730

[18] Bonomi AE Wagner EH Glasgow RE VonKorff M. Assessment of Chronic Illness Care (ACIC): a practical tool to measure quality improvement. Health Services Res. (2002) 37:791–820. 10.1111/1475-6773.00049 12132606 PMC1434662

[19] Mazzucca S Parks RG Tabak RG Allen P Dobbins M Stamatakis KA. Assessing organizational supports for evidence-based decision making in local public health departments in the United States: development and psychometric properties of a new measure. J Public Health Manage Pract. (2019) 25:454–63. 10.1097/PHH.0000000000000952 31348160 PMC6614014

[20] Zimmerman L Lounsbury DW Rosen CS Kimerling R Trafton JA Lindley SE. Participatory system dynamics modeling: increasing stakeholder engagement and precision to improve implementation planning in systems. Adm Policy Ment Health. (2016) 43:834–49. 10.1007/s10488-016-0754-1 27480546 PMC8344379

[21] Adam T. Advancing the application of systems thinking in health. Health Res Policy Sys. (2014) 12:1478. 10.1186/1478-4505-12-50 25160646 PMC4245197

[22] Diez Roux AV. Complex systems thinking and current impasses in health disparities research. Am J Public Health. (2011) 101:1627–34. 10.2105/AJPH.2011.300149 21778505 PMC3154209

[23] Ovretveit J. Understanding the conditions for improvement: research to discover which context influences affect improvement success. BMJ Q Safety. (2011) 20:i18–i23. 10.1136/bmjqs.2010.045955 21450764 PMC3066695

[24] May CR Johnson M Finch T. Implementation, context and complexity. Implementation Sci. (2016) 11:141. 10.1186/s13012-016-0506-3 27756414 PMC5069794

[25] Kemp CG Wagenaar BH Haroz EE. Expanding hybrid studies for implementation research: intervention, implementation strategy, and context. Front Public Health. (2019) 7:325. 10.3389/fpubh.2019.00325 31781528 PMC6857476

[26] Wiltsey Stirman S Baumann AA Miller CJ. The FRAME: an expanded framework for reporting adaptations and modifications to evidence-based interventions. Implementation Sci. (2019) 14:58. 10.1186/s13012-019-0898-y 31171014 PMC6554895

[27] Chambers DA Norton WE. The adaptome: advancing the science of intervention adaptation. Am J Prev Med. (2016) 51:S124–31. 10.1016/j.amepre.2016.05.011 27371105 PMC5030159

[28] Baumann AA Cabassa LJ. Reframing implementation science to address inequities in healthcare delivery. BMC Health Serv Res. (2020) 20:190. 10.1186/s12913-020-4975-3 32164706 PMC7069050

[29] Haire-Joshu D Hill-Briggs F. The next generation of diabetes translation: a path to health equity. Ann Rev Public Health. (2019) 40:391–410. 10.1146/annurev-publhealth-040218-044158 30601723

[30] Warne D Frizzell LB. American Indian health policy: historical trends and contemporary issues. Am J Public Health. (2014) 104:S263–7. 10.2105/AJPH.2013.301682 24754649 PMC4035886

[31] Office of Urban Indian Health Programs. Uniform Data System Summary Report Final - 2017. (2021). Available online at: https://www.ihs.gov/sites/urban/themes/responsive2017/display_objects/documents/2017_UIHP_UDS_Summary_Report_Final.pdf (accessed September 9, 2021).

[32] Fleischhacker S. Emerging opportunities for registered dietitian nutritionists to help raise a healthier generation of Native American Youth. J Acad Nutr Dietetics. (2016) 116:219–25. 10.1016/j.jand.2015.10.018 26680608 PMC4733391

[33] O'Connell J Reid M Rockell J Harty K Perraillon M Manson S. Patient outcomes associated with utilization of education, case management, and advanced practice pharmacy services by American Indian and Alaska Native peoples with diabetes. Med Care. (2021) 59:477–86. 10.1097/MLR.0000000000001521 33758159 PMC8609964

[34] Garcia AN Castro MC Sánchez JP. Social and structural determinants of Urban American Indian and Alaska Native health: a case study in Los Angeles. MedEdPORTAL. (2019) 15:mep_2374-8265.10825. 10.15766/mep_2374-8265.10825 31161137 PMC6543927

